# High-Molecular-Weight Polyphenol-Rich Fraction of Black Tea Does Not Prevent Atrophy by Unloading, But Promotes Soleus Muscle Mass Recovery from Atrophy in Mice

**DOI:** 10.3390/nu11092131

**Published:** 2019-09-06

**Authors:** Yuki Aoki, Tetsuo Ozawa, Osamu Numata, Tohru Takemasa

**Affiliations:** 1 Graduate School of Comprehensive Human Sciences, University of Tsukuba, 1-1-1 Tennodai, Tsukuba 305-8574, Japan; 2 Graduate School of Life and Environmental Sciences, University of Tsukuba, 1-1-1 Tennodai, Tsukuba 305-8572, Japan (T.O.) (O.N.)

**Keywords:** black tea, polyphenol, hindlimb suspension, reloading, mTOR

## Abstract

Previously, we reported that polyphenol-rich fraction (named E80) promotes skeletal muscle hypertrophy induced by functional overload in mice. This study indicates that E80 has potential for affecting skeletal muscle mass. Then, we evaluate the effect of E80 on atrophic and recovery conditions of skeletal muscle in mice. Hindlimb suspension (unloading) and relanding (reloading) are used extensively to observe disuse muscle atrophy and subsequent muscle mass recovery from atrophy. Eight-week old C57BL/6 mice were fed either a normal diet or a diet containing 0.5% E80 for two weeks under conditions of hindlimb suspension and a subsequent 5 or 10 days of reloading. We found that E80 administration did not prevent atrophy during hindlimb suspension, but promoted recovery of slow-twitch (soleus) muscle mass from atrophy induced by hindlimb suspension. After five days of reloading, we discovered that phosphorylation of the Akt/mammalian target of rapamycin (mTOR) pathway proteins, such as Akt and P70 ribosomal protein S6 kinase (S6K), was activated in the muscle. Therefore, E80 administration accelerated mTOR signal and increased protein synthesis in the reloaded soleus muscle.

## 1. Introduction

Tea beverage such as green tea and black tea comprises leaves of *Camellia sinensis*. Its history is extensive and people in Asia have used tea for about 4000 years [1,2]. Recent studies indicate that low-molecular-weight tea polyphenol, such as catechins and theaflavins, have various beneficial effects. Concerning the effects of tea polyphenols on skeletal muscle, they improve endurance capacity [3], glucose uptake [4], and reduction of Endoplasmic reticulum (ER) stress [5]. They also play a part in the prevention of disuse skeletal muscle atrophy [6,7]. These reports suggest that tea polyphenol can be a candidate for improving skeletal muscle metabolism and maintaining muscle mass.

Skeletal muscle is a highly plastic tissue and responds well to environmental and physiological stimulations [8]. Overloading or unloading alter skeletal muscle mass, which is determined by the rates of both protein synthesis and degradation. Higher rates of protein degradation compared with those of synthesis within muscle result in a decrease in muscle mass (atrophy). On the other hand, higher rates of protein synthesis compared with those of degradation stimulate an increase in muscle mass (hypertrophy) [9]. Unloading, such as inactivity or space flight, causes disuse muscle atrophy in humans [10,11]. In rodents, the hindlimb unloading (HU) model is used extensively to observe disuse muscle atrophy, including molecular events [12]. The HU model is also useful in evaluating recovery responses from atrophy by relanding its legs (reloading (RE)) [12]. 

Muscle atrophy is characterized by decreases in muscle mass, the myofiber cross-sectional area (CSA) [13,14]. Subsequent mechanical reloading induces muscle regrowth. Importantly, disuse muscle atrophy, induced by such as inactivity or spaceflight, is a crucial clinical matter because impaired skeletal muscle strength can extend the duration of hospital care and result in exercise limitation, as well as spoil quality of life (QOL) [15]. Furthermore, the potential to prevent recovery of muscle mass after atrophy is of great clinical importance because impaired or retarded muscle regrowth can have severe consequences for functional performance [16,17]. Thus, prevention of muscle atrophy and acceleration of muscle mass recovery are indispensable for maintaining QOL. 

Previously, we reported that high-molecular-weight polyphenol-rich fraction (“E80”) from black tea promotes hypertrophy and activates the mammalian target of rapamycin (mTOR) signal, which regulates protein synthesis within functional overloaded skeletal muscle in mice [18]. This result indicates that E80 increases skeletal muscle mass and also stimulates protein synthesis with mechanical overload. Thus, we assessed whether E80 prevents muscle atrophy induced by HU or urges recovery of atrophied muscle mass induced by mechanical reloading.

## 2. Results

### 2.1. E80 Has No Effect on Food Intake and Growth

**Figure 1:**
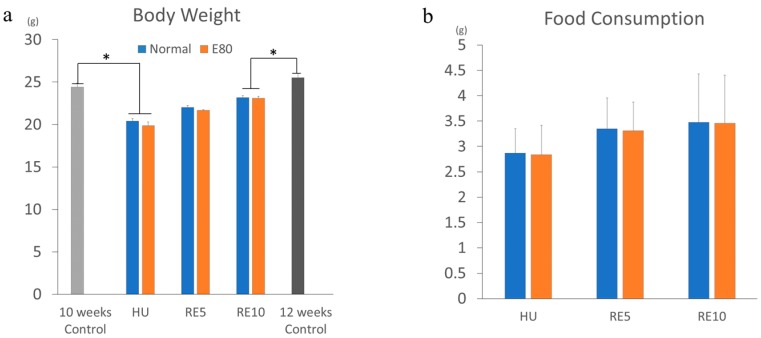
E80 has no effect on food intake and growth. Body weight change during hindlimb unloding (HU) and reloading (**a**); and food consumption per day (**b**) between hindlimb unloding (HU), 5-day or 10-day of reloading (RE5, RE10), and control (10 weeks or 12 weeks control). Values are presented as mean ± SEM (*n* = 6–7). * indicates significant difference (*p* < 0.05).

We used E80 extracted from the same batch of the previous report; the component of E80 is shown there [18]. The extracts were stored in dark conditions at less 20 °C, 50% humidity for 10 months. The feature of E80 is a slight amount of caffeine (0.38%) (Figure S1). A high dose of caffeine causes severe symptoms [19]; therefore, tea extract containing caffeine is unsuitable for utilization as a supplement. We performed the same experiment twice during this period. The contents of E80 were considered to be exceedingly consistent for this duration, because Shiming Li et al. demonstrated that tea extracts are very stable under conditions of room temperature (27 °C) and 60% humidity [20]. They showed that tea polyphenols such as epigallocatechin gallate (EGCG) and theaflavin are not oxidized or degraded for 360 days at conditions of 27 °C and 60% humidity [20]. Flavanol glycosides are also consistent in dark conditions [21]. Hence, we consider that contents of E80 were the same as those in the previous study. As we reported previously [18], there was no difference in body weight between Normal and E80 among all groups (HU, RE5, and RE10) (Figure 1a). There was also no difference in food consumption between Normal and E80 among all groups (HU, RE5, and RE10) (Figure 1b). These data show that E80 intake alone does not affect food consumption and body growth, as we reported previously.

### 2.2. E80 Does Not Prevent HU-Induced Muscle Atrophy

**Figure 2:**
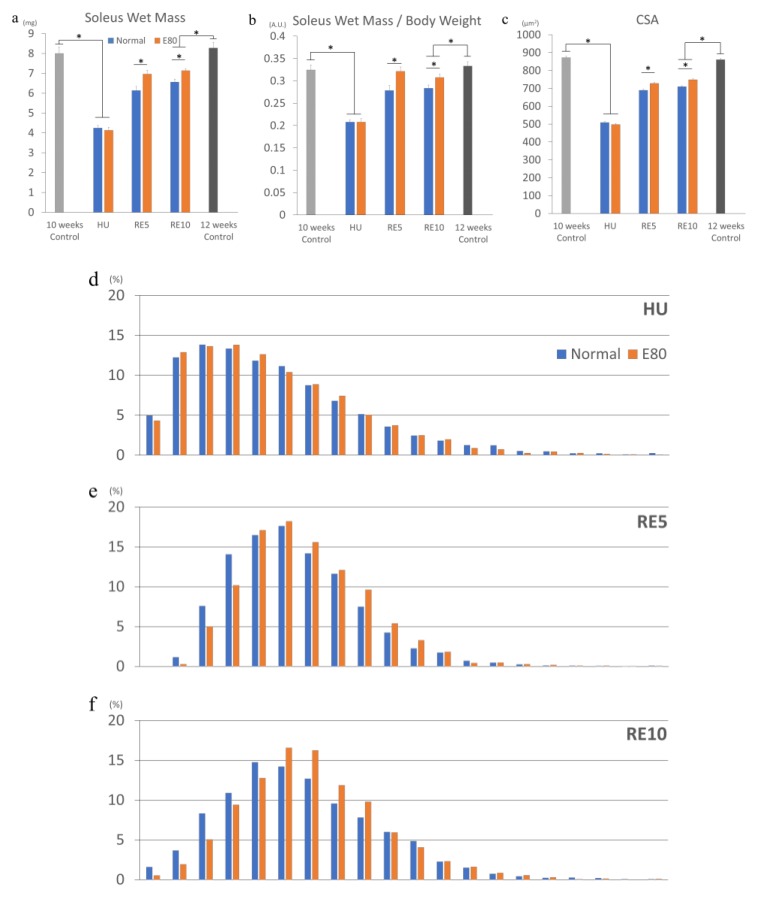

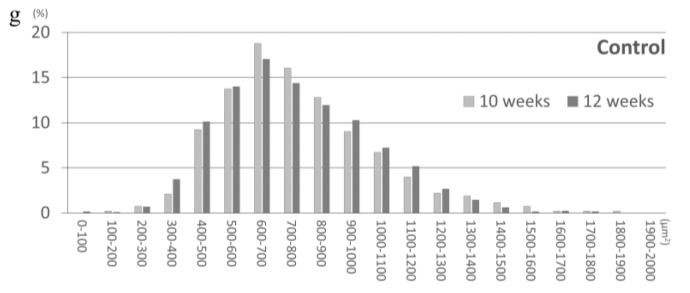
E80 did not prevent HU-induced atrophy, but promoted recovery of muscle mass. Muscle wet mass (**a**), muscle wet mass/body weight (**b**), cross-sectional area (**c**), and distribution of fibers (**d**–**g**) of soleus muscle of HU, RE5, RE10, and control groups (**g**). * indicates significant difference (*p* < 0.05) (*n* = 6–7). CSA, cross-sectional area.

In order to evaluate the effect of E80 on HU-induced atrophy, we compared the wet mass of soleus muscle, the wet weight of soleus muscle/body weight, and the CSA of soleus muscle between the Normal and the E80 of the HU group, because HU exerts the greatest atrophic effects on antigravity muscles such as the soleus muscle [12,22]. The wet mass of soleus reduced drastically by approximately 50% compared with the 10-week control (equivalent age to HU groups) after two weeks of HU, as previously reported [12,23] (Figure 2a). However, there was no difference between Normal and E80 in the HU group (Figure 2a). There were also no changes in the soleus wet mass/body weight ratio in the HU group (Figure 2b). Concerning the CSA, thickness of the fibers significantly decreased in both Normal and E80 of the HU group, compared with the 10-week control (equivalent age to HU groups), but no significant difference was observed between Normal and E80 (Figure 2c). In addition, the shape of the fiber distribution was quite similar between Normal and E80 of the HU group (Figure 2d). These results suggest that E80 had no effect on preventing disuse muscle atrophy and on body weight reduction during HU.

### 2.3. E80 Promotes Muscle Mass Recovery from HU-Induced Atrophy

Subsequently, we analyzed reloaded RE5 and RE10 groups. The soleus wet mass of the RE5 and RE10 groups increased compared with the HU group (Figure 2a). In particular, the muscle mass of E80 of RE5 and RE10 significantly boosted. Similarly, the soleus wet mass/body weight ratio and CSA regained higher compared with those of the HU group (Figure 2b,c). Moreover, administration of E80 promoted recovery of the wet mass/body weight ratio and the CSA compared with Normal in the RE5 and RE10 groups (Figure 2b,c). Consistent with the CSA data, fiber distribution of the E80 group of RE5 (hereafter, described as RE_E80) was shifted rightward. In particular, the number of fibers above 700 µm^2^ was further increased compared with RE5_Normal (Figure 2e). As RE5, E80 of RE10 group has thicker fibers (800 to 900 µm^2^, above average) than Normal (Figure 2f). These data indicate that administration of E80 promoted the restoration of skeletal muscle mass from disuse muscle atrophy induced by HU. To be certain, we compared mice between age at beginning (10 weeks old) and at the end (12 weeks old) of this experiment, and the CSA and fiber distribution between the two groups were almost the same (Figure 2c,g).

### 2.4. E80 Activates the Akt/mTOR Pathway During Reloading

Skeletal muscle hypertrophy, an increase in skeletal muscle mass and CSA, is considered to be a result of enhanced protein synthesis. The major pathway regulating protein synthesis in skeletal muscle is the insulin-like growth factors (IGF)/mTOR signal [9]. Phosphorylation of Akt encourages protein synthesis in the skeletal muscle [9]. Moreover, phosphorylated Akt promotes the activation of mTOR and its downstream targets, such as P70 ribosomal protein S6 kinase (S6K) and eukaryotic translation initiation factor 4E-binding protein 1 (4EBP1) [9]. In summary, activation of Akt leads to escalated protein synthesis in skeletal muscle.

**Figure 3:**
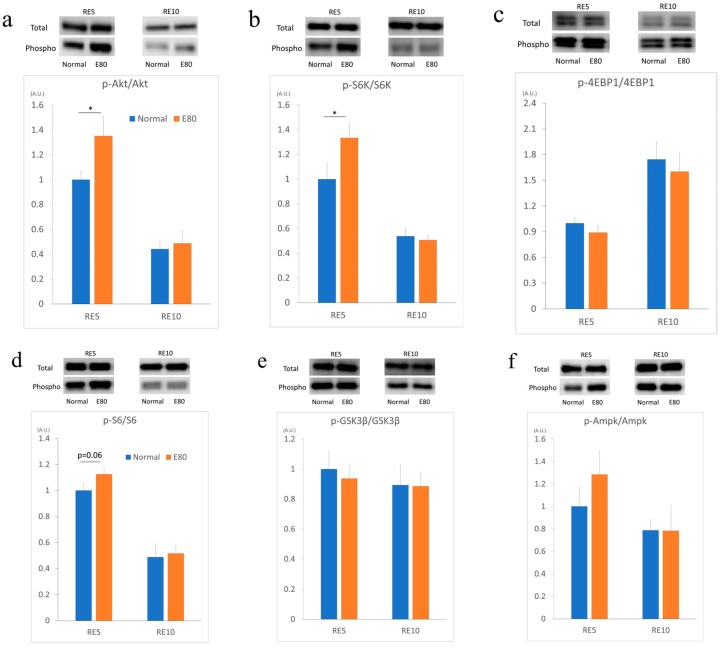
E80 activated Akt/mTOR pathway signaling. Western blot bands of total and phosphorylated protein (above) and relative ratio of phosphorylated protein/total protein (below) of soleus muscle proteins using antibodies against p-Akt (thr) and Akt (**a**), p-P70 ribosomal protein S6 kinase (S6K) and S6K (**b**), p-eukaryotic translation initiation factor 4E-binding protein 1 (4EBP1) and 4EBP1 (**c**), p-S6 and S6 (**d**), p-glycogen synthase kinase 3 beta (GSK3b) and GSK3b (**e**), and p-5′ adenosine monophosphate-activated protein kinase (Ampk) and Ampk (**f**). Soleus muscle was prepared on the RE5 and RE10 groups. Quantitative data represent mean ± SEM. * indicates significant difference (*p* < 0.05) (*n* = 6–7).

The recovery processes of muscle mass during reloading are similar to those that occur after resistance exercise, as both excite protein synthesis in skeletal muscle and result in enhancement of muscle mass. Therefore, we measured the factors of the mTOR signal. First, we analyzed the level of Akt phosphorylation in the soleus muscle of RE5 and RE10. In the RE5 group, the phosphorylation level of Akt of RE5_E80 was significantly increased (Figure 3a). Next, we evaluated the phosphorylation levels of S6K and 4EBP1. In the RE5 group, the phosphorylation level of S6K significantly increased in the soleus muscle of RE5_E80 (Figure 3b), but that of 4EBP1 did not increase (Figure 3c). As S6 is a direct target of S6K [24], the phosphorylation level of S6 was measured. Similar to that of S6K, the phosphorylation level of S6 was greater than in RE5_E80 in the soleus muscle, but not significantly (*p* = 0.06) (Figure 3d). In regard to other factors, glycogen synthase kinase 3 beta (GSK3β) and 5′ adenosine monophosphate-activated protein kinase (Ampk), no change was observed in the phosphorylation levels (Figure 3e,f). These data indicate that E80 intake promotes the phosphorylation of Akt, S6K in reloaded soleus muscle in the RE5 group. In the RE10 group, there are no differences between Normal and E80 groups in all six proteins.

### 2.5. E80 Stimlated S6K and S6 Phospholyration In Vitro

**Figure 4:**
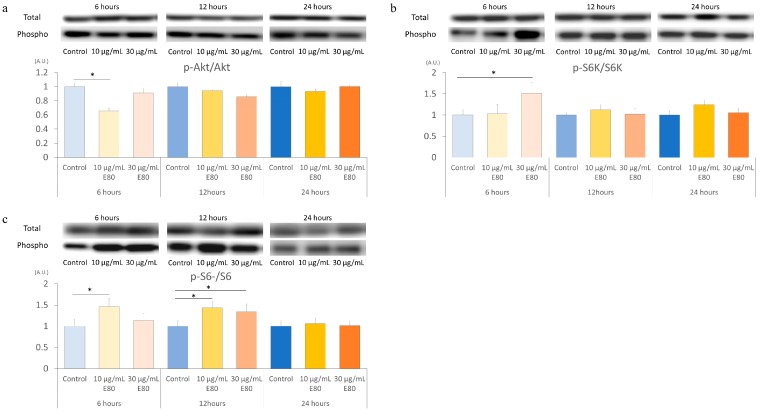
E80 stimulated S6K and S6 phosphorylation in vitro. C2C12 myotubes treated with 10 µg/mL or 30 µg/mL of E80. Western blot bands of total and phosphorylated protein (above) and relative ratio of phosphorylated protein/total protein (below) of differentiated C2C12 myotubes proteins using antibodies against p-Akt (thr) and Akt (**a**), p-S6K and S6K (**b**), and p-S6 and S6 (**c**). Quantitative data represent mean ± SEM. * indicates significant difference (*p* < 0.05) (*n* = 5–6).

Finally, we evaluated whether E80 has the potential to affect skeletal muscle cell directly in vitro. We used cultured skeletal muscle cell C2C12 myoblasts. Differentiated C2C12 forms myotubes were treated with two different E80 concentrations (10 µg/mL or 30 µg/mL) for 6 h, 12 h, and 24 h. Firstly, we analyzed the level of Akt phosphorylation in C2C12 myotubes. Akt phosphorylation level declined in myotubes treated with 10 µg/mL E80 for 6 h (Figure 4a). The Akt phosphorylation in myotubes treated with 30 µg/mL E80 did not change compared with that in the 6 h control group (Figure 4a). There is no difference of Akt phosphorylation level in 12 h or 24 h group between the control and E80 groups (Figure 4a). Next, we evaluated the phosphorylation levels of S6K in myotubes. The phosphorylation level of S6K significantly increased in myotubes treated with 30 µg/mL E80 for 6 h (Figure 4b). However, that in myotubes treated with 10 µg/mL E80 did not change (Figure 4b). There is no difference in 12 h or 24 h group between the control and E80 groups (Figure 4b). Concerning the phosphorylation level of S6, that in myotubes treated with 10 µg/mL E80 for 6 h was greater than that for 6 h control (Figure 4c). Similarly, those in myotubes treated with 10 µg/mL and 30 µg/mL for 12 h increased compared with that in 12 h control (Figure 4c). However, these differences disappeared in the 24 h groups (Figure 4c). 

## 3. Discussion

We observed that supplementation of E80 does not prevent muscle atrophy induced by HU (Figure 2a–d), but it promotes recovery of muscle mass and the phosphorylation of Akt and S6K when reloading (Figure 3a,b).

E80 administration promoted recovery of the soleus wet mass, soleus wet mass/body weight ratio, and the CSA compared with those in Normal. However, these indicators did not restore to the level of those in the 12-week control (equivalent age to RE10). As previously reported, the soleus wet mass, soleus wet mass/body weight ratio did not recover to the control level after 7 days of reloading, but they recovered after 14 days of reloading [17,25]. In addition, the CSA did not recover to the control level after 14 days of reloading, but they recovered after 28 days of reloading [17,25]. The reason that these indicators did not return to basal control level is that the mice body weight did not recover to basal control level after several days. This occurred because stimulation of hindlimb muscle is a mechanical load from the body weight of mice. In this experiment, mice body weight also did not recover to basal control during the 10 days of reloading (Figure 1a).

Akt is a crucial regulator of skeletal muscle hypertrophy [26]. Akt induces activation of mTOR, which activates S6K [26]. Furthermore, the levels of phosphorylated S6K correlate with the magnitude of skeletal muscle hypertrophy after resistance training [26]. Akt and S6K were significantly activated in RE5_E80 compared with RE5_Normal. Additionally, although it is not significant, S6 was seemingly activated. According to these results, E80 promoted protein synthesis during reloading and resulted in a fast recovery of muscle mass from HU-induced atrophy compared with Normal. Additionally, dominant-negative Ampk transgenic mice in which a dominant-negative Ampkα2 subunit was overexpressed under the muscle creatine kinase promoter (expressed in heart and skeletal muscle) showed that EDL muscles tended to be larger than in wild-type mice, suggesting that Ampk might negatively regulate basal muscle mass [27]. However, in this experiment, we did not observe activation of Ampk (Figure 3f).

The phosphorylation level of Akt, S6K, and S6 did not differ between E80 and Normal in the RE10 group. Some reports showed that the phosphorylation level of these proteins reached the highest after approximately three days of reloading and declined gradually until it returned to normal. It is possible that these proteins were activated in RE10_E80 if they were analyzed during days 2 to 3 of reloading. In vitro experiment, the phosphorylation level of Akt decreased with 10 µg/mL of E80 for the 6 h treatment group (Figure 4a). However, the phosphorylation level of S6K seemed to be activated with 30 µg/mL of E80 for the 6 h treatment group (Figure 4b). Likewise, the phosphorylation level of S6 with 10 µg/mL of E80 for the 6 h treatment group was increased, as well as those with 10 µg/mL and 30 µg/mL of E80 for the 12 h treatment groups (Figure 4c).

Administration of E80 did not prevent muscle atrophy induced by HU. We previously assessed whether E80 prevented denervation-induced muscle atrophy. Similar to this experiment, E80 did not block the reduction of muscle mass (Figure S1a,b). We also analyzed the mRNA expression level of ubiquitin ligase regulating muscle atrophy, such as Murf1 and MAFbx, during one week of denervation, but these gene mRNA expression levels did not change between Normal and E80 (Figure S1c,d). However, in this experiment, we showed that E80 promoted recovery of muscle mass during reloading. Also, E80 stimulated the phosphorylation level of Akt and S6K after five days of reloading. In addition to in vivo experiments, E80 also activated S6K or S6 in C2C12 myotubes (Figure 4b,c). Depending on the concentration or duration of treatment, E80 increased the phosphorylation level of these proteins. In vitro and in vivo experiments are not comparable simply, but we think E80 has the potential for increasing phosphorylation levels of S6K and S6, and encouraging muscle mass recovery and could be a useful supplemental candidate for restoring skeletal muscle mass after inactivity or injury. Also, it may play a role in human skeletal muscle development.

It is not accessible to activate S6K of skeletal muscle in elderly men with resistance training, because S6K phosphorylation does not increase the rate of protein synthesis in elderly men compared with younger men [28]. These facts indicate that it is difficult for elderly men to enhance signal transmission in the cell and to increase protein synthesis. The main cause of sarcopenia is considered to be the gradual loss of muscle mass and function [29,30]. If E80 promotes S6K phosphorylation and protein synthesis in aged people, it may be an optimal supplement for maintaining or restoring muscle mass caused by sarcopenia. For practical utilization of E80 as a supplement for both elderly men and athletes, further studies to appraise the effect of E80 on aged mice or aged humans are important.

## 4. Materials and Methods

### 4.1. HU and Reloading

All animal experiment procedures were performed in accordance with the institutional guidelines for the care and use of laboratory animals as approved by the University of Tsukuba (No 18-357). Animals were subjected to HU according to a previously described method [31]. Male C57BL/6J mice were utilized (Charles River Laboratories Japan, Kanagawa, Japan). Mice were maintained at 22~25 °C, and kept on a 12/12-h light/dark cycle. Mice were housed in cylindrical enclosures (18 cm in diameter). At day 0, mice assigned to the HU group (*n* = 7) were unloaded. Under isoflurane anesthesia (2%~3%), bandage tapes were braided around the tail with a paper clip and a swivel hook. The metal string was thread through the swivel hook and fixed to the top of the cage to suspend the hindlimbs of the mice. Mice toes were kept lifted about 3 cm from the bottom in order that their feet not touch the cage floor. Each suspended mouse could move freely, using its forelimbs over the entire floor area of the cage. After two weeks of HU, mice were released from suspension and their hindlimb muscles were reloaded. Food and water were available ad libitum during the entire experimental period. Mice were allowed to acclimate to their cages for one week before experiments commenced. After the prescribed procedure, mice were anesthetized with isoflurane and subsequently sacrificed. The soleus muscle was carefully dissected, wet mass measured immediately, and frozen in liquid nitrogen. They were stored at −80 °C until use.

### 4.2. Cell Culture and Treatment

All cell culture experiments were performed in a humidified environment at 37 °C in 5% CO_2_. The skeletal muscle cell lineage C2C12 myoblasts were proliferated in Dulbecco’s modified eagle’s medium (DMEM) (Life Technologies Corporation, New York, United States of America) supplemented with 10% fetal bovine serum (FBS) and penicilin-streptomicin up to 50% confluency in six-well plate (BM Equipment Co., Ltd., Tokyo, Japan). When cells reached 80% confluency, serum content in culture medium was switched from 10% FBS to 2% horse serum so as to induce the differentiation of myoblasts into myotubes. After seven days of differentiation, myotubes were stimulated with each concentration of E80 (10 µg/mL and 30 µg/mL) for 6 h, 12 h, and 24 h. E80 was dissolved in 100% dimethyl sulfoxide (DMSO), of which the final concentration was prepared for 0.1% in serum free DMEM. The series of experiments was repeated two times using different passages of C2C12 myotubes.

### 4.3. Preparation and Storage of E80 from Black Tea

Black tea (30 g; Daily club; Mitsui Norin Co., Ltd., Tokyo, Japan) was seethed for 1 min in boiling water (1000 mL), and then allowed to stand for 10 min. The brew was filtered using double-layered filter paper (number 2; Advantec, Toyo Roshi Kaisha, Tokyo, Japan). The filtrate was mixed with 250 mL Toyopearl HW-40F (Tosoh, Tokyo, Japan) previously washed with water. After 30 min, Toyopearl HW-40F was filtered using a filter paper and washed 10 times with 150 mL of water. Polyphenolic substances adsorbed to the Toyopearl resin were extracted 12 times with 150 mL of 80% (*v/v*) warm ethanol. All extracts were evaporated under reduced pressure and freeze dried to yield 2.96 g. This dark brown powder was named E80. The sample was stored in the dark and under 20 °C until use.

### 4.4. Quantitative Analysis of Caffeine, Catechins, and Theaflavins in E80

High performance liquid chromatography (HPLC) analysis was executed using the Inertsil ODS-3 column (4.6 × 250 mm, 5 µm; GL Sciences, Tokyo, Japan) attached to a Shimadzu Class M10A HPLC system (Shimadzu, Kyoto, Japan). The column was eluted at 40 °C with a linear gradient from 5% to 40% (*v/v*) acetonitrile containing 0.02% (*v/v*) trifluoroacetic acid over 70 min at a flow rate of 0.7 mL/min and monitored for absorbance at 280 nm. Quantitative determination was based on the calibration curve prepared with an adequate concentration of a pure commercial standard of caffeine, catechins, and theaflavins. Samples were dissolved in 10% (*v/v*) acetonitrile and 4.0 µL portions were injected into the column.

### 4.5. Quantitative Analysis of Highly Polymerized Polyphenols in E80

For preparation of the analytical sample from E80, 382 mg of E80 was dissolved in water (100 mL). The solution was extracted eight times with 40 mL ethyl acetate to remove the ethyl acetate-soluble constituents. The water phase was evaporated under reduced pressure to remove the ethyl acetate in the solution and the pH was adjusted to 3 with hydrochloric acid (HCl). The solution was then extracted five times with 40 mL n-butanol. The aqueous phase of n-butanol was concentrated under reduced pressure at 50 °C and freeze dried. The yield was 140 mg.

For quantitative analysis of the highly polymerized polyphenols in E80, we used the following analytical instruments that consisted of two medium-pressure SP-11 delivery pumps (Tokyo Rika Kikai, Tokyo, Japan), a gradient mixer, a sample injector VI-II (EYELA), an intermediate pressure glass column (1 × 30 cm) packed with Toyopearl HW-40F, and a fraction collector CHF161RA (Advantec, Tokyo, Japan). The analytical sample (1.7 mL) was dissolved in 20% (*v/v*) acetone and injected into the column, which was conditioned with 20% acetone. The column was eluted using a linear gradient containing from 20% to 50% acetone (total volume 180 mL) at a flow rate of 0.4 mL/min. The eluent (1.5 g) was collected by a fraction collector and the absorbance was measured at 350 nm. From the elution profile, the highly polymerized polyphenol fractions were combined, evaporated under reduced pressure, freeze dried, and then weighed.

### 4.6. Diet Preparation

Mice of the normal diet group were fed with a powdered diet (NMF; Oriental yeast Co., Tokyo, Japan), and those in the E80 group were fed with NMF containing 0.5% E80. Seven groups were prepared: non-treated mice eating only NMF (control), unloaded mice for two weeks eating only NMF (HU_Normal), unloaded mice eating NMF mixed with E80 (HU_E80), reloaded mice for five days eating only NMF (RE5_Normal), reloaded mice for five days eating NMF including E80 (RL5_E80), reloaded mice for 10 days eating only NMF (RE10_Normal group), and reloaded mice for 10 days eating NMF including E80 (RE10_E80 group).

### 4.7. Antibodies

We used the following antibodies: anti-Akt (No. 9272), anti-phospho-Akt (Ser473; No. 9271), anti-S6K (No. 9202), anti-phospho-S6K (Thr389; No. 9205), anti-S6 (No. 2217), anti-phospho-S6 (Ser235/236; No. 4858), anti-4EBP1 (No. 9644), anti-phospho-4EBP1 (Thr37/46; No. 2855), anti-GSK3β (No. 12456), anti-phospho-GSK3β (Ser9; No. 5558), anti-Ampk (No. 2532), anti-phospho-Ampk (Ser9; No. 2535), anti-mouse IgG (No. 7076), and anti-rabbit IgG (No. 7074) purchased from Cell Signaling Technology (Massachusetts, United States of America). Anti-α-Tubulin (12G10) was purchased from DSHB (Iowa, United States of America). Anti- Laminin α-2 (SC-59854) was purchased from Santa Cruz Biotechnology (Texas, United States of America).

### 4.8. CSA Quantification

Dissected soleus muscle was soaked in optimal cutting temperature compound (Sakura Finetek, Tokyo, Japan), and then immediately frozen in isopentane iced with liquid nitrogen and stored at −30 °C until sectioning. Frozen muscle samples were sectioned at a thickness of 12 μm, air dried, and stored at −30 °C. Images were captured with the BZ-X710 microscope (Keyence, Osaka, Japan).

To verify the CSA of muscle fibers, the muscle sections were incubated overnight with Laminin alpha2 antibody to stain the sarcolemma.

Subsequently, the sections were incubated with Alexa for 90 min and then washed with Tris-buffered saline with Tween 20 (TBS-T, 50 mM Tris (pH = 7.4), 138 mM NaCl, 2.7 mM KCl, and 0.05% Tween 20) three times for 5 min. The stained sections were observed under the BZ-X710 microscope and CSA analysis was carried out using the BZ-X Analyzer (Keyence).

### 4.9. Western Blot Analysis

To prepare total protein lysate, frozen muscle samples were homogenized in lysis buffer (1% Nonidet-P40, 1% sodium deoxycholate, 0.2% sodium dodecyl sulfate(SDS), 150 mM NaCl, 50 mM 4-(2-hydroxyethyl)-1-piperazineethanesulfonic acid (HEPES) (pH = 7.5), 10 mM ethylenediaminetetraacetic acid (EDTA), 10 mM NaF, 10 mM Na_4_P_2_O_7_, and 2 mM Na_3_VO_4_) supplemented with 1% protease inhibitor cocktail for mammalian tissues (No. 162-0177; Nacalai Tesque, Kyoto, Japan). The concentration of protein was determined by the bicinchoninic acid method using the Protein Assay Bicinchoninate Kit (No. 297-73101, Wako, Osaka, Japan). Protein extracts were electrophoresed on appropriate concentration (7.5%–15.0%) of acrylamide gels and subsequently transferred to polyvinylidene difluoride membranes (PVDF; No. 162-0177; Bio-Rad, California, United States of America). The PVDF membranes were blocked with 5% skim milk in Tris Buffered Saline with Tween 20 (TBS-T) for 45 min at room temperature (20~25 °C) and then incubated overnight with primary antibody at 4 °C. Subsequently, the membrane was incubated with anti-rabbit IgG for 90 min. The intensity of proteins was evaluated using the LI-COR system (No. CDG002134) and quantified by Image Studio Digits 4.0 software (Nacalai Tesque).

### 4.10. Statistical Analysis

A one-way analysis of variance (ANOVA) was performed using SPSS software (IBM Corp., New York, United States of America) to determine whether a significant interaction existed between the two independent factors.

## Supplementary Materials

The following are available online at https://www.mdpi.com/2072-6643/11/9/2131/s1, Figure S1: E80 did not prevent denervation-induced atrophy and gene expression concerning muscle atrophy.

## Abbreviations

4EBP1	Eukaryotic translation initiation factor 4E-binding protein 1
Ampk	5′ adenosine monophosphate-activated protein kinase
ANOVA	Analysis of variance
CSA	Cross sectional area
DMEM	Dulbecco’s modified eagle’s medium
DMSO	Dimethyl sulfoxide
EDTA	Ethylenediaminetetraacetic acid
EGCG	Epigallocatechin gallate
ER	Endoplasmic reticulum
FBS	Fetal bovine serum
GSK3β	Glycogen synthase kinase 3 beta
HEPES	4-(2-hydroxyethyl)-1-piperazineethanesulfonic acid
HPLC	High performance liquid chromatography
IGF	Insulin-like growth factors
mTOR	Mammalian target of rapamycin
S6K	P70 ribosomal protein S6 kinase
SDS	Sodium dodecyl sulfate
PVDF	Polyvinylidene difluoride
S6	Ribosomal protein S6 kinase
