# Supplementary material for: High-Molecular-Weight Polyphenol-Rich Fraction of Black Tea Does Not Prevent Atrophy by Unloading, But Promotes Soleus Muscle Mass Recovery from Atrophy in Mice
Source: Nutrients. 2019 Sep 6;11(9):2131. doi: 10.3390/nu11092131 (PMC6770236; doi:10.3390/nu11092131)
Supplement: Supplementary file 1 [file nutrients-11-02131-s001.zip › nutrients-576213-supplementary.pptx]

## Slide 1
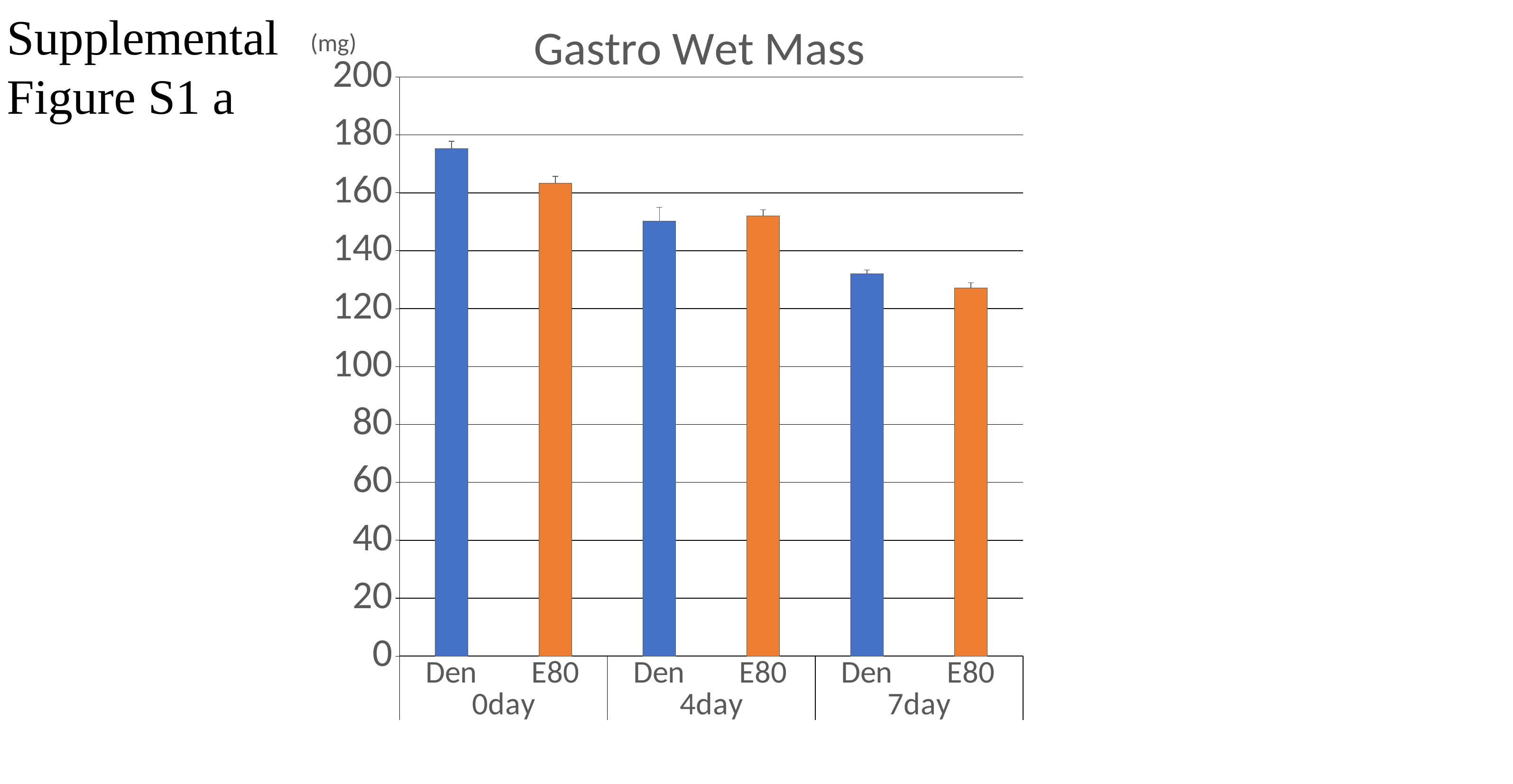

### Chart: Gastro Wet Mass
| Category | |
|---|---|
| Den | 175.22222222222223 |
| E80 | 163.29444444444445 |
| Den | 150.30555555555557 |
| E80 | 152.07222222222222 |
| Den | 131.97500000000002 |
| E80 | 127.22222222222223 |Supplemental Figure S1 a
(mg)

## Slide 2
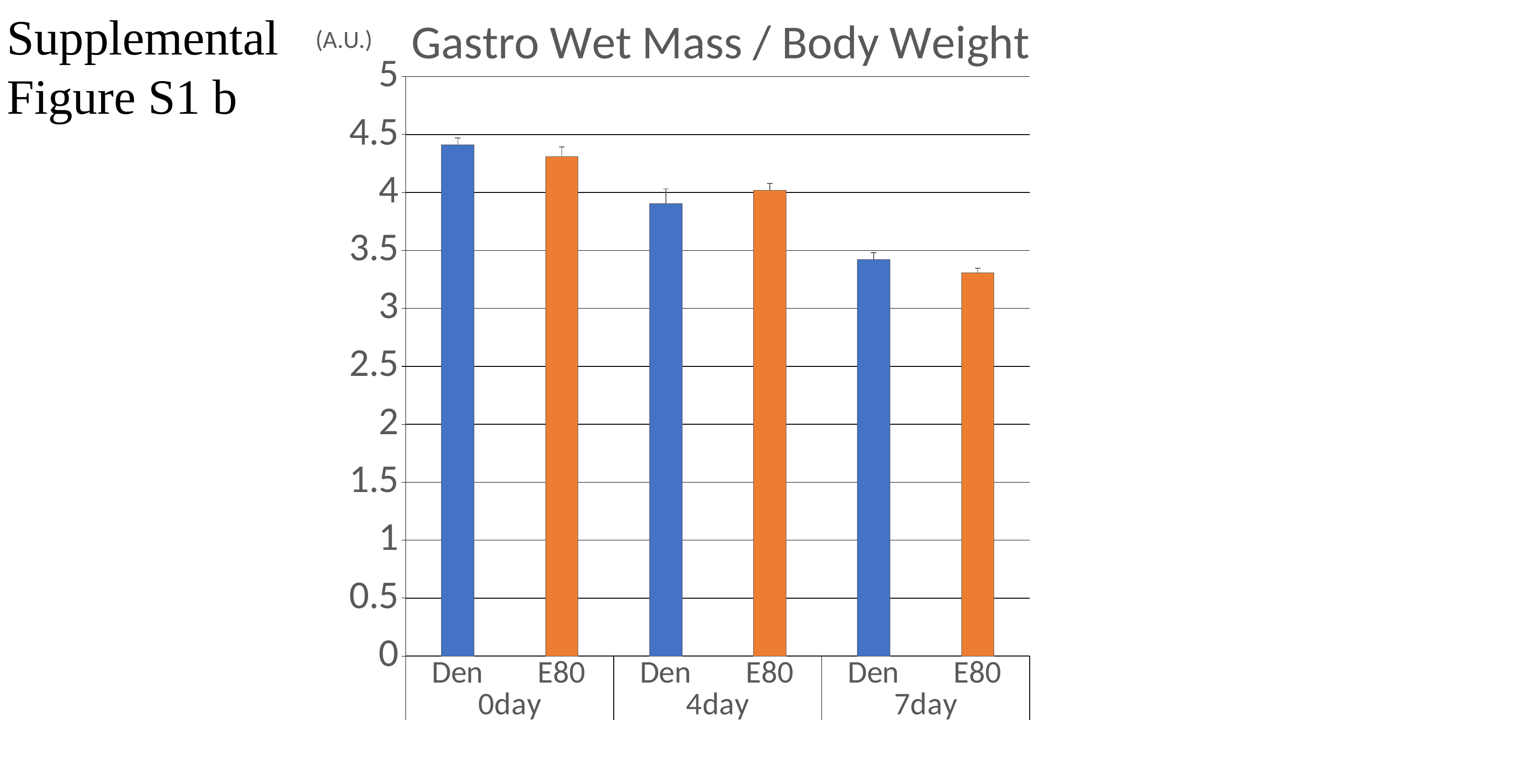

### Chart: Gastro Wet Mass / Body Weight
| Category | |
|---|---|
| Den | 4.412156306666679 |
| E80 | 4.309212799123886 |
| Den | 3.906031699197936 |
| E80 | 4.018613871624777 |
| Den | 3.42366493877275 |
| E80 | 3.307066854536783 |Supplemental Figure S1 b
(A.U.)

## Slide 3
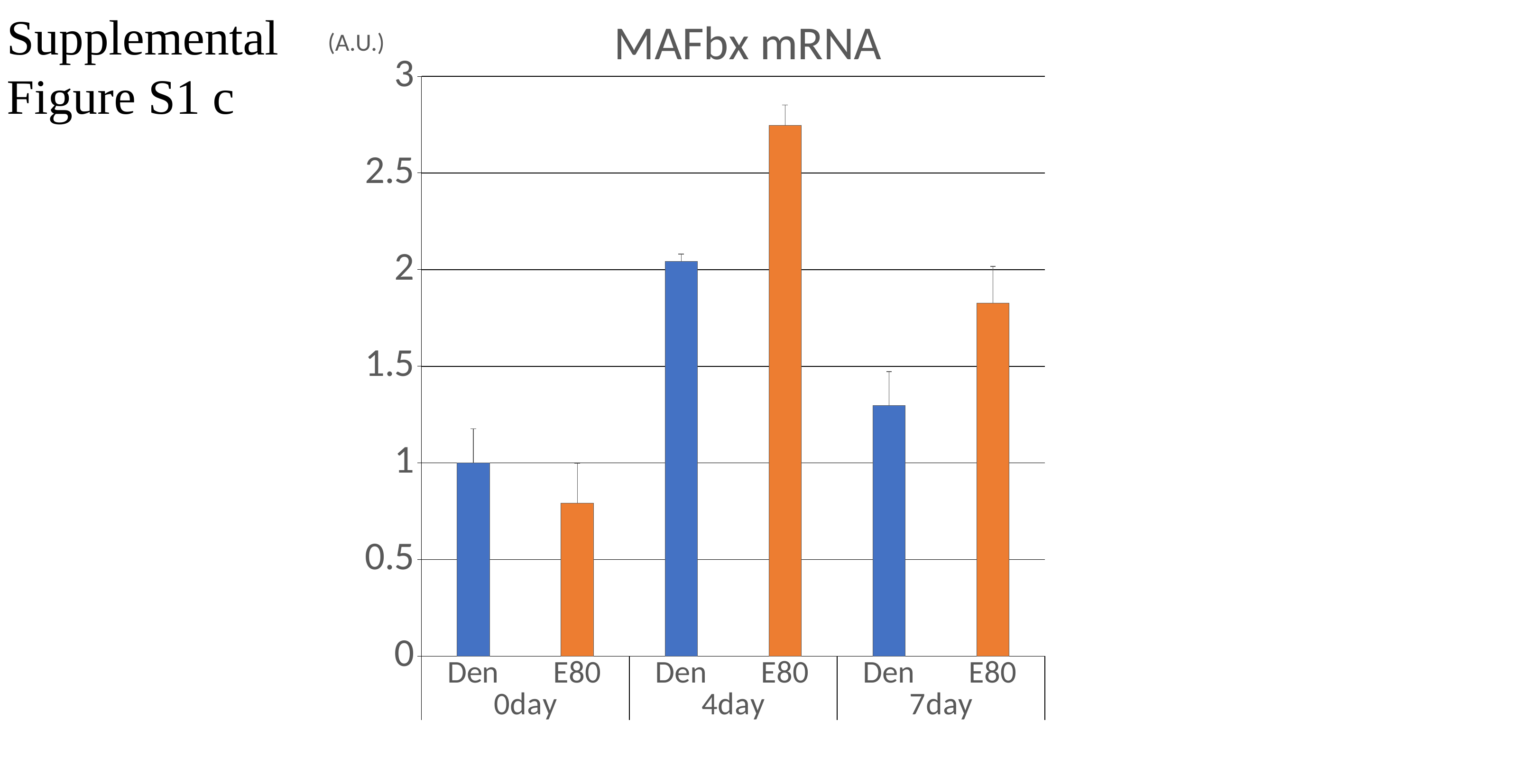

### Chart: MAFbx mRNA
| Category | |
|---|---|
| Den | 1.0 |
| E80 | 0.7918688052797198 |
| Den | 2.042024251414387 |
| E80 | 2.7463204997497885 |
| Den | 1.298338588161578 |
| E80 | 1.8276629004588019 |Supplemental Figure S1 c
(A.U.)

## Slide 4
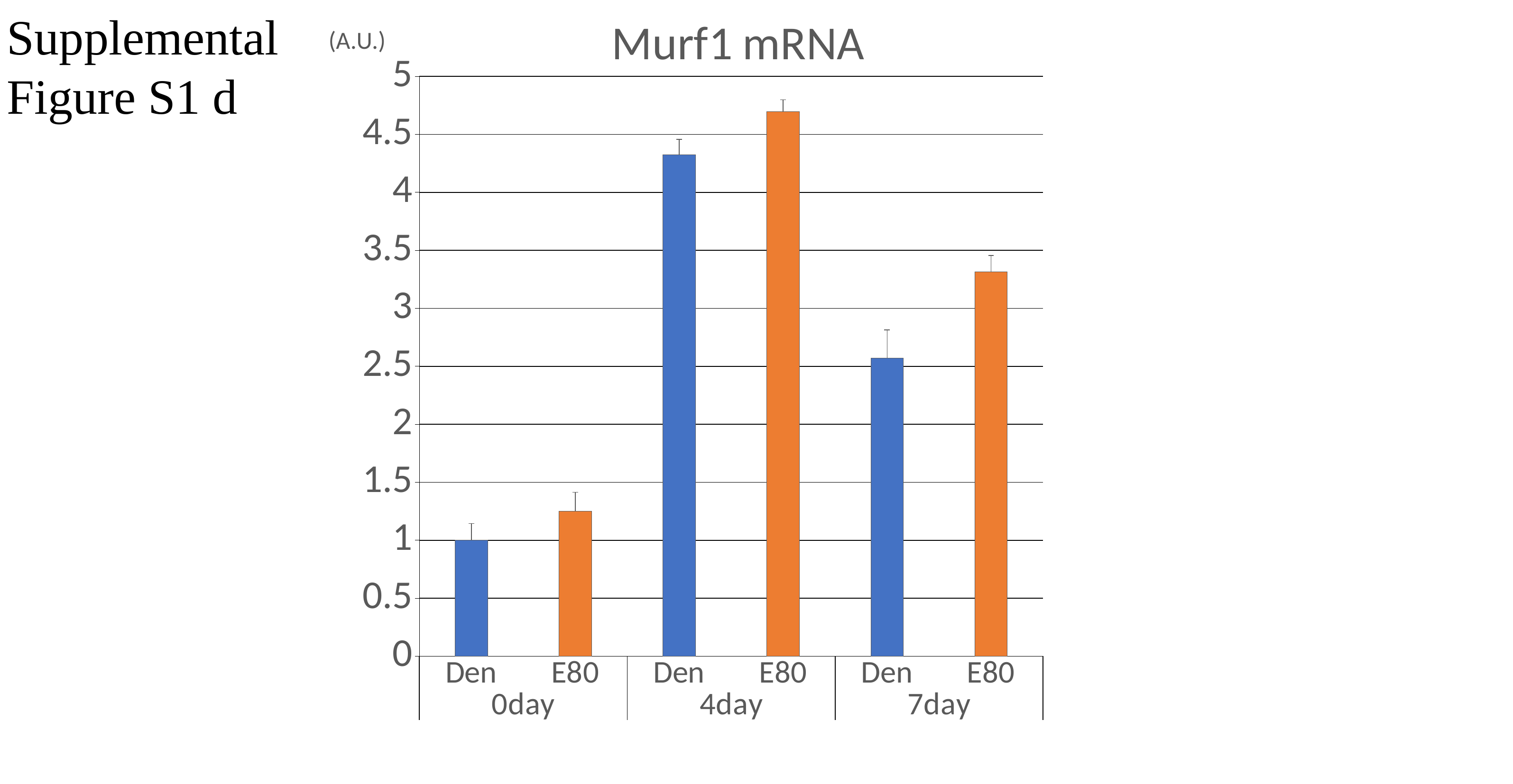

### Chart: Murf1 mRNA
| Category | |
|---|---|
| Den | 1.0000000000000004 |
| E80 | 1.2512181394937494 |
| Den | 4.32190292118085 |
| E80 | 4.696762576900441 |
| Den | 2.5698188521096568 |
| E80 | 3.3134481259784727 |Supplemental Figure S1 d
(A.U.)
